# Lepidopteran Synteny Units reveal deep chromosomal conservation in butterflies and moths

**DOI:** 10.1093/g3journal/jkad134

**Published:** 2023-06-13

**Authors:** Walther Traut, Ken Sahara, Richard H ffrench-Constant

**Affiliations:** Institut für Biologie, Zentrum für Medizinische Struktur- und Zellbiologie, Universität zu Lübeck, Ratzeburger Allee 160, D-23562 Lübeck, Germany; Laboratory of Molecular Entomology, Faculty of Agriculture, Iwate University, 3-18-8, Ueda, Morioka 020-8550, Japan; Centre for Ecology and Conservation, University of Exeter, Penryn Campus, Penryn, UK

**Keywords:** chromosome evolution, macrosynteny, Lepidoptera, chromosome numbers, chromosome rearrangement, BUSCO

## Abstract

DNA is compacted into individual particles or chromosomes that form the basic units of inheritance. However, different animals and plants have widely different numbers of chromosomes. This means that we cannot readily tell which chromosomes are related to which. Here, we describe a simple technique that looks at the similarity of genes on each chromosome and thus gives us a true picture of their homology or similarity through evolutionary time. We use this new system to look at the chromosomes of butterflies and moths or Lepidoptera. We term the associated synteny units, Lepidopteran Synteny Units (LSUs). Using a sample of butterfly and moth genomes from across evolutionary time, we show that LSUs form a simple and reliable method of tracing chromosomal homology back through time. Surprisingly, this technique reveals that butterfly and moth chromosomes show conserved blocks dating back to their sister group the Trichoptera. As Lepidoptera have holocentric chromosomes, it will be interesting to see if similar levels of synteny are shown in groups of animals with monocentric chromosomes. The ability to define homology via LSU analysis makes it considerably easier to approach many questions in chromosomal evolution.

## Introduction

Chromosome number varies widely in animals and plants, from *n* = 1 in the nematode *Parascaris univalens* (Boveri 1899; Goday and Pimpinelli 1986) and the ant *Myrmecia pilosula* (Crosland and Crozier 1986) to *n* = 720 in the fern *Ophioglossum reticulatum* (Khandelwal 2008). In animals, chromosome number also differs markedly between taxonomic groups. Within the insects, for example, Diptera (the true flies) have small numbers of chromosomes showing little variation, whereas Orthoptera (the grasshoppers) have higher numbers of chromosomes. It is, however, far from clear what rules might govern chromosomal evolution between groups of species. There is, for example, no simple overall correlation between total genomic DNA content and chromosome number. The evolution of chromosome numbers might be driven by the need to link favorable combinations genes in synteny. This hypothesis based on the idea of chromosomal territories in the interphase nucleus (Cremer and Cremer 2001, Meaburn and Misteli 2007) where genes of potentially similar function are kept in close contact with each other in such territories.

To examine any potential biological role of chromosome diversity in genome evolution, we need to be able to trace the ancestry of individual chromosomes. However, due to ambiguity in the prior literature, it is first necessary to state what exactly we mean by the term synteny. We use synteny in its originally defined sense, meaning literally “on the same ribbon” (Renwick 1971). This meaning is therefore the physical equivalent of the genetic term “linkage,” which has no specific regard to gene order. In turn, we use the term “macrosynteny” to refer to large conserved syntenic blocks which may encompass whole chromosomes. Finally, in this study we use “conserved gene order,” or “collinearity,” to detect intrachromosomal rearrangements. With these definitions in mind, we developed a simple homology-based technique for exploring conserved synteny in the butterflies and moths. The Lepidoptera have previously been shown to have wide-ranging synteny or macrosynteny across autosomes and Z chromosomes via a range of different mapping techniques including comparative linkage mapping (Pringle *et al*. 2007; Beldade *et al*. 2009; Baxter *et al*. 2011; Van't Hof *et al*. 2013) and BAC-FISH mapping (Yasukochi *et al*. 2009; Yoshido *et al*. 2011; Sahara *et al*. 2013) by a comparison of assemblies and of annotated long-read sequences (d’Alençon *et al*. 2010; The Heliconius Genome Consortium 2012) and by whole-genome comparison (Höök *et al*. 2023, Pazhenkova and Lukhtanov 2023).

Currently, the genomes of more than 200 lepidopteran species have been fully sequenced with chromosome-scale assembly, mainly by the Darwin Tree of Life Consortium (https://www.darwintreeoflife.org). These complete insect genomes therefore now afford a deeper analysis of macrosyteny across the different phylogenetic branches of the butterflies and moths. We selected 13 of these complete genomes representing species from different branches of the lepidopteran phylogeny plus 1 species of the sister order Trichoptera. To compare synteny across distantly related species, we cannot rely on direct comparison of chromosomes and genomes, for example, by Dotplot analysis (e.g. GEPARD, Krumsiek *et al*. 2007). Instead, we used Benchmarking Universal Single-Copy Orthologs (BUSCO) (Manni *et al*. 2021). BUSCO analysis is classically used in genomics to determine the apparent completeness of a genome by comparing the observed vs the expected number of protein coding sequences predicted (Feron and Waterhouse 2022). However, here we use BUSCO-derived scores containing the positional information for each BUSCO marker to compare the location of these markers in the chromosomes of different species. In this respect, the 5,286 BUSCO gene markers in the lineage-specific collection “lepidoptera_odb10” present an unprecedented density of markers for all lepidopteran genomes. This comparative BUSCO analysis thus allows us to compare genomes from the most basal phylogenetic branch of the Lepidoptera to the more derived ones and even to a representative of a different insect order, the Trichoptera. Here, we show that butterfly and moth chromosomes are made up of well-conserved synteny units, which we term Lepidopteran Synteny Units (LSUs). These LSUs are conserved in a wide phylogenetic spectrum of species including the basal group of Micropterigidae and the representative of the sister group Trichoptera. This means that the genome of ancestral Lepidoptera in the late Triassic period of geology was already organized into these basic macrosynteny units. We argue that such a homology-based system advances our understanding of how chromosomes evolve and that such a system avoids the confusion generated by simply comparing chromosome numbers.

## Materials and methods

### Lepidopteran Synteny Units

The model species *Melitaea cinxia* was chosen as a reference point for interspecies comparisons as it has a well-characterized genome and karyotype with the presumed ancestral number of 31 chromosomes (Ahola *et al*. 2014, Traut *et al*. 2017). We ran BUSCO version 5.4.3 (https://busco.ezlab.org/) with the lineage data set “lepidoptera_odb10” (https://busco.ezlab.org/list_of_lineages.html) on the *M. cinxia* genome. The BUSCO output “full_table.tsv” presents the positional information of all 5,286 lepidoptera_odb10 markers, line by line. We discarded entries noted “Missing” or “Duplicated.” The remaining 5.210 marker lines were sorted according to the chromosome and the respective chromosomal position in the *M. cinxia* genome. The 31 chromosome-specific marker subsets define what we call LSUs (Supplementary Table 1). LSU_31 is the Z chromosomal subset of markers. For easier use in subsequent comparisons, we constructed a table named “LSU_1-31_table” that contained all LSU markers, ordered according to LSU and chromosome position.

### Analysis of LSUs in different lepidopteran species

**Fig. 1.**
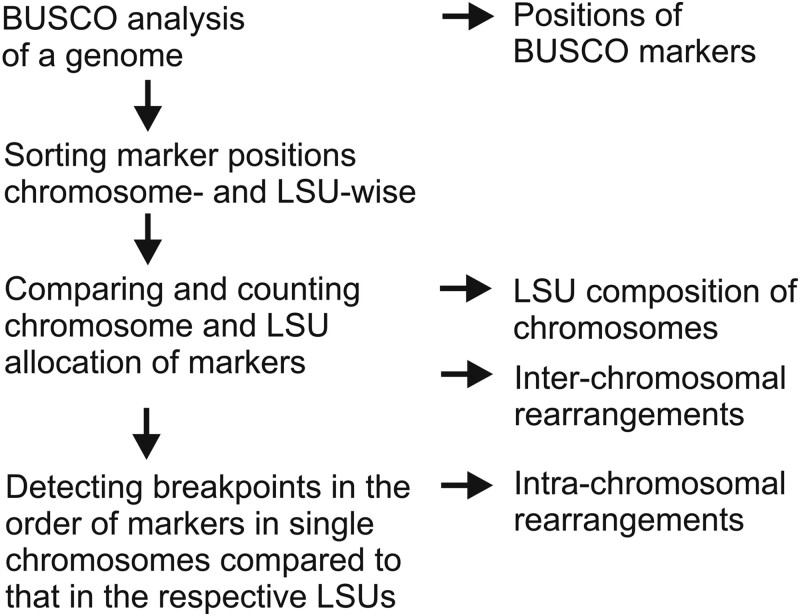
Workflow of LSU analysis.

In order to compare LSU marker positions across the Lepidoptera phylogeny, we ran a BUSCO analysis with the lepidoptera_odb10 marker set on each target genome. The BUSCO output “full_table.tsv” was then combined line by line with LSU_1-31_table using the application “BUSCO_output_to_LSUs.java” (LSU table and code available at figshare, doi:10.6084/m9.figshare.22672996). The combined table as well as a table of hit counts for all LSUs can be saved in.tsv format and further processed in a spreadsheet program. Rearranging the order of lines according to the sorted chromosome column thus presented us with the composition of each target chromosome. To determine and count intrachromosomal rearrangements in the target chromosomes relative to the model *M. cinxia* (LSUs), we compared and searched the marker positions for breakpoints in the order of markers. LSUs and target chromosomes were compared with “LSU_breakpoints.java” (available at figshare, doi:10.6084/m9.figshare.22673005). The whole process of LSU analysis is summarized in Fig. 1. Details of the 13 lepidopteran genomes and the trichopteran genome chosen for our analysis, accession, and chromosome numbers are compiled in Supplementary Table 2. Finally, we note that a recent publication (Pazhenkova and Lukhtanov 2023) described synteny relationship among the satyrine butterflies *Maniola jurtina*, *Erebia aethiops*, and *Erebia ligea* using a different method (Minimap2, Li 2018). For an independent validation of our procedure, we ran a BUSCO analysis on the same genomes and reassuringly, found precisely the same relationships among them (Supplementary Table 3).

## Results

### Marker coverage

We chose the model butterfly *M. cinxia* as an anchor genome as it has both a published chromosome-scale genome (Vila *et al*. 2021) and the inferred ancestral chromosome number of Lepidoptera, where *n* = 31 (Ahola *et al*. 2014, Traut *et al*. 2017). Using BUSCO (Manni *et al*. 2021), we mapped 5210 of the 5,286 markers from the lepidoptera_odb10 set to the *M. cinxia* genome. These markers covered most of the length of all chromosomes (Table 1). The marker subsets specific for the 31 chromosomes and autosomes plus Z chromosome were then used to investigate the extent of conserved synteny in the other selected species. They exposed a similar breadth of coverage and well-conserved blocks of synteny which we term LSUs. Supplementary Table 1 lists the respective 31 LSU marker subsets of lepidoptera_odb10.

**Table 1.** Coverage of *M. cinxia* chromosomes with BUSCO markers.

		Coverage		
	Size (Mbp)	from pos.	to pos.	Number of markers
Chr1	20.85	117,417	19,685,649	297
Chr2	20.73	398,625	20,236,179	297
Chr3	20.52	726,777	20,152,629	315
Chr4	19.96	238,280	19,835,936	215
Chr5	19.01	483,178	18,560,843	276
Chr6	18.77	1,353,307	18,709,134	200
Chr7	18.41	495,890	18,396,783	184
Chr8	17.99	31,643	17,951,792	224
Chr9	17.92	708,144	15,949,575	225
Chr10	17.88	119,154	17,419,592	223
Chr11	17.73	297,087	17,078,688	158
Chr12	17.33	113,696	17,026,419	175
Chr13	17.30	610,143	16,190,007	136
Chr14	17.10	20,325	16,394,340	230
Chr15	17.03	157,748	16,520,130	188
Chr16	16.95	89,162	16,836,400	154
Chr17	16.66	664,664	16,611,164	192
Chr18	16.15	438,756	15,719,455	185
Chr19	15.58	168,057	15,511,538	111
Chr20	14.99	1,475,615	14,762,925	163
Chr21	14.42	65,125	14,259,913	169
Chr22	13.79	91,040	13,623,835	76
Chr23	12.93	75,696	12,806,138	121
Chr24	12.54	1,191,162	12,464,274	137
Chr25	12.17	665,594	12,001,985	42
Chr26	11.88	802,033	11,124,055	53
Chr27	11.31	481,414	11,189,652	45
Chr28	10.93	438,482	9,922,115	66
Chr29	9.05	45,181	8,887,344	72
Chr30	8.85	239,960	8,588,656	47
ChrZ	22.67	150,007	22,438,970	234

### LSUs and chromosome numbers

**Fig. 2.**
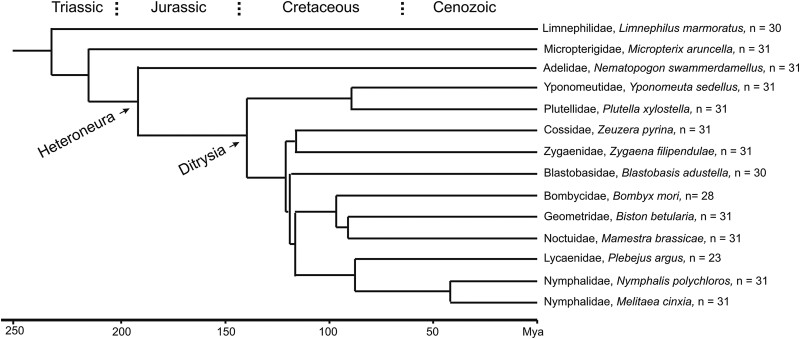
Phylogeny of the 13 lepidopteran species and the trichopteran species *L. marmoratus* studied in this paper. The phylogeny is based on data from Malm *et al*. (2013), Wahlberg *et al*. (2013), and Chazot *et al*. (2019).

For our phylogenetic analysis of chromosome composition and synteny, we selected 12 other Lepidoptera genomes and 1 from the order Trichoptera. The species were chosen among those with available chromosome-scale genomes to represent different branches of the lepidopteran phylogeny (Fig. 2). BUSCO searches in the selected genomes revealed nearly chromosome-sized blocks of conserved syntenic markers Lepidoptera-wide and even extending to Trichoptera (Supplementary Table 4). Chromosome-wide macrosynteny (irrespective of marker gene order) is almost perfect among the chromosomes of the 12 heteroneuran Lepidoptera species examined here. These 12 species are from 11 different families: Nymphalidae, Lycaenidae, Noctuidae, Geometridae, Bombycidae, Blastobasidae, Zygaenidae, Cossidae, Plutellidae, Yponomeutidae, and Adelidae, and therefore represent a broad cross section of the clade Heteroneura. This clade comprises more than 99% of the ∼160,000 lepidopteran species (Kristensen 1999).

**Fig. 3.**
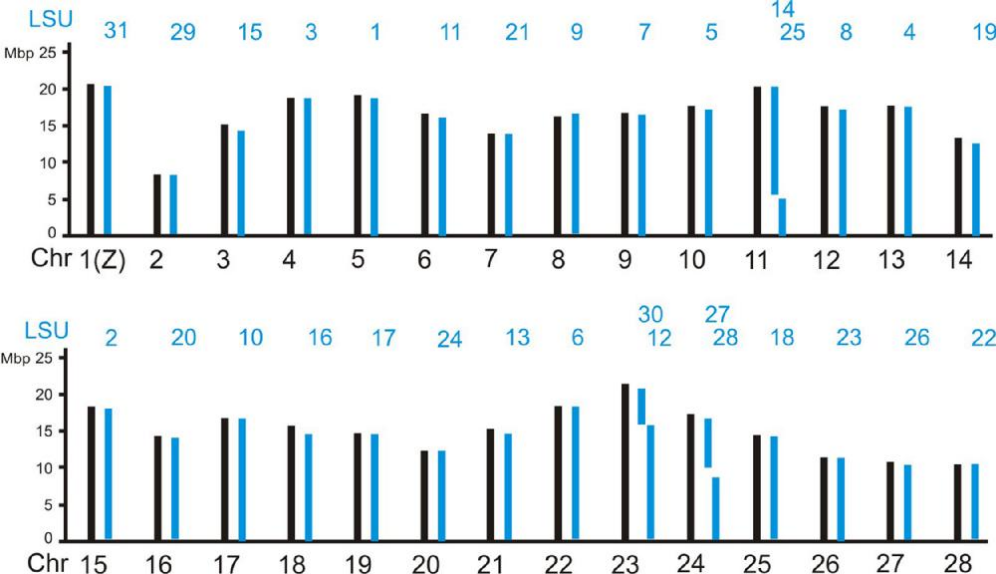
LSU composition of the 28 chromosomes from *B. mori* (Bombycidae).

**Fig. 4.**
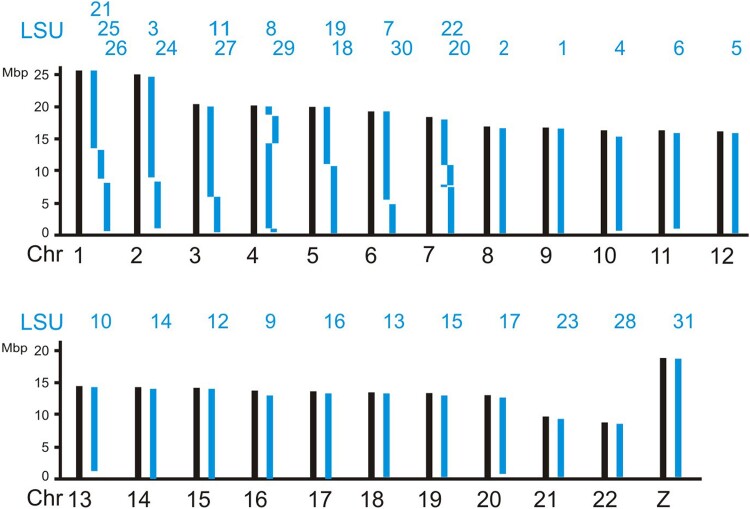
LSU composition of the 23 chromosomes from *P. argus* (Lycaenidae).

Haploid chromosome numbers (autosomes plus Z chromosome) range from *n* = 23 to *n* = 31 among the heteroneuran species examined here. The representatives of the families Nymphalidae (*M. cinxia* and *Nymphalis polychloros*), Noctuidae (*Mamestra brassicae*), Geometridae (*Biston betularia*), Plutellidae (*Plutella xylostella*), Cossidae (*Zeuzera pyrina*), Yponomeutidae (*Yponomeuta sedellus*), and Adelidae (*Nematopogon swammerdamellus*) have sets of *n* = 31 chromosomes. Each of the chromosomes constitutes 1 of the 31 LSUs defined here (Supplementary Table 4). The representatives of the 4 remaining heteroneuran families have less than 31 chromosomes in their haploid sets of chromosomes. *Zygaena filipendulae* from the family Zygaenidae has *n* = 30 chromosomes. Supplementary Table 4 shows that its chromosome 21 is a fusion product of 2 LSUs, LSU_25, and LSU_27. In *Blastobasis adustella* from the family Blastobasidae, another species with *n* = 30 chromosomes, it is the Z chromosome that is a fusion product of 2 LSUs, in this case LSU_21 and the original Z chromosomal unit LSU_31. The silkworm *Bombyx mori* from the Bombycidae family has *n* = 28 chromosomes. Three of the chromosomes, chromosomes 11, 23, and 24 can be seen composed of 2 LSUs each (Fig. 3), confirming previous results (The *Heliconius* Genome Consortium 2012, Yasukochi *et al*. 2016). The *n* = 23 chromosomes of *Plebejus argus* from the family Lycaenidae are the result of even more fusions, chromosome 1 consists of 3 LSUs, chromosomes 2–7 of 2 LSUs each (Fig. 4). In summary, the 31 LSUs or 31 blocks of conserved synteny are an ancestral character in the clade of Heteroneura represented by those families. The situation is slightly different in *Micropterix aruncella* and *Limnephilus marmoratus*, as discussed further below.

### Chromosomal rearrangements

Having detected deep synteny across the order Lepidoptera, we then wanted to examine the frequency of inter- and intrachromosomal rearrangements, such as inversions and translocations, via changes in the observed order of LSU markers. We selected 4 species with different divergence times for a study of such rearrangements, namely, *Nymphalis polychlorus*, *B. mori*, *Y. sedellus*, and *N. swammerdamellus*, each compared with *M. cinxia*. No large interchromosomal translocations, either reciprocal or nonreciprocal, were detected. Small nonreciprocal transpositions of single or 2–3 adjacent markers happened at a low frequency of 0.01–0.27 per Mbp, 4–135 events per genome and, as expected, increased with divergence time (Table 2). In contrast, intrachromosomal rearrangements were frequent. For a measure, we counted the breakpoints in the order of markers. Inversions leave 2 breakpoints and translocations 3 breakpoints, as footprints in the rearranged chromosome. As expected, the number of breakpoints also increased with divergence time, from 96 to 1,665 per genome. This corresponds to a rate of 0.46–3.34 per Mbp. It is important to note, however, that the resolution of rearrangement detection is limited by the density of available markers. The actual rate of rearrangements is presumably therefore much higher. For example, this analysis loses all rearrangements that can only be seen at the nucleotide level, such as those detected by comparisons of Pacific Biosciences derived long-read sequences from closely related species (d’Alençon *et al*. 2010).

**Table 2.** Intra- and interchromosomal rearrangements detected as breakpoints in the order of LSU markers.

	Breakpoints		Transposition of LSU markers			
Species	within LSUs	per Mbp	single	groups of 2–3	per Mbp	Time of divergence
*N. polychloros*	232	0.46	4	0	0.01	∼42 My
*B. mori*	468	0.94	15	1	0.03	∼116 My
*Y. sedellus*	639	1.28	32	2	0.07	∼139 My
*N. swammerdamellus*	1665	3.34	135	1	0.27	∼172 My

Rearrangements registered in 4 species relative to *M. cinxia*. Time of divergence between *M. cinxia* (LSUs) and the target species according to Wahlberg *et al*. (2013) and Chazot *et al*. (2019).

### *M. aruncella* and *L. marmoratus* as basal entities

Lepidopteran macrosynteny extends to, but is less stringently conserved in, the more distantly related species *M. aruncella*, which is from the basal lepidopteran family Micropterigidae and in *L. marmoratus* from the family Limnephilidae of the sister order Trichoptera (Supplementary Table 4). Fully conserved are 30 of the 31 LSUs in either 1 or the other of the 2 species and 24 still intact in the trichopteran *L. marmoratus*. But more genes are transferred to other chromosomes, and a few larger interchromosomal rearrangements were documented by the mapped LSU markers. One of the synteny blocks, LSU_2, is conserved in all heteroneuran species but is split in *M. aruncella* and *L. marmoratus*. This means, in fact, that LSU_2 is a fusion product of 2 chromosomes. The fusion must have taken place during evolution of Lepidoptera after the branching off of Micropterigidae and before diversification of the Heteroneura. The other interchromosomal rearrangements may have taken place in the respective lineages. LSU_10 is entire in *M. aruncella* but split in *L. marmoratus* where it forms Chr27 and part of Chr10. In *L. marmoratus*, LSU_29 and LSU_30 together form Chr26. The fate of LSU_18 in *M. aruncella* is a remarkable case of chromosome instability. While it was intact in the trichopteran species as well as in all heteroneuran species, it contributed to 28 of the 31 chromosomes of *M. aruncella*, almost always in small fractions of the unit, 1–9 of 129 markers. The only major contribution, 44 of the 129 markers, was made to the Z chromosome, where it joined the original Z chromosome block, LSU_31.

## Discussion

### Lepidopteran Synteny Units and chromosome number

Here we show that lepidopteran genomes are composed of well-conserved blocks of synteny which we term Lepidopteran Synteny Units or LSUs. There are 31 of them in the lepidopteran clade Heteroneura which contains more than 99% of the ∼160,000 butterfly and moth species. Each LSU defines the composition of a single chromosome in the representative species with karyotypes of *n* = 31 chromosomes. In those with less chromosomes, some chromosomes are composed of 2 or 3 LSUs combined (see Figs. 2 and 3). The analysis of the trichopteran *L. marmoratus* showed that 24 of the LSUs already existed as chromosomal entities before Lepidoptera and Trichoptera lineages split, some 230 million years ago (Mya) (Wiegmann *et al*. 2009). This estimate of age may indeed be too conservative. According to Kawahara *et al*. (2019) the oldest members of crown Lepidoptera lived ∼300 Mya. The full set of LSUs came into being after Micropterigidae branched off from the main stem of Lepidoptera and before the diversification of Heteroneura, about 172 Mya (Wahlberg *et al*. 2013), or ∼230 Mya according to Kawahara *et al*. (2019).

The modal number of chromosomes in Lepidoptera is 31 according to Robinson (1971). The occurrence of species with *n* = 31 chromosomes, each represented by a single LSU, in Adelidae, Yponomeutidae, Cossidae, Plutellidae, Nymphalidae, Geometridae, and Noctuidae confirms that *n* = 31 is the ancestral karyotype in the Heteroneura clade of Lepidoptera. Karyotypes with a different chromosome number are derived from it. The ancestral chromosome number at the root of Lepidoptera, however, cannot yet be deduced. Although the micropterigid *M. aruncella* has *n* = 31 chromosomes, the same number, some of the chromosomes are not homologous to the 31 chromosomes of Heteroneura. In this species as well as in the trichopteran *L. marmoratus*, LSU_2 is found split into 2 chromosomes. Thus the number should have been 32, but disruption of 1 LSU and fusions of others prohibit a conclusion (for details see Results). Sequencing another species of this group may clarify this picture.

### Sex chromosomes

The Z chromosomes of all species studied here share the same LSU, LSU_31. This means that Lepidoptera and Trichoptera have the same Z chromosome, as prviously noted by Fraïsse *et al*. (2017). In *B. adustella,* the ancestral Z chromosome, represented by LSU_31, is fused with an autosome, represented by LSU_21 and in *M. aruncella* the original Z chromosome is enlarged by an originally autosomal chunk from LSU_18. In a few of the selected species, the female restricted W chromosomes were sequenced, however they are not included here as no BUSCO hits were recorded on them.

### Rearrangements within and between LSUs

We have shown that LSUs are highly conserved across the Heteroneura and, moreover, large rearrangements between LSUs are not found within our chosen species spanning this group. Gains and losses of LSUs therefore reflect a more subtle pattern of “leakage” whereby individual LSUs are present in some species and absent in others. However, this pattern is not ubiquitous. A few “hot spots’ of interchromosomal exchange were observed within the sampled genomes. LSU_18 in *M. aruncella* is nearly pulverized except for 44 of the 129 markers enlarging the Z chromosome of this species, 85 of the 129 markers were contributed to various other chromosomes. Not quite as dramatic, LSU_28 in *P. xylostella* contributed 41 of 55 markers to Chr_31 and 14 to other chromosomes. It will be interesting to investigate what causes the instability in these chromosomes now that we have a tool to examine their homology.

### Synteny in other taxa and future applications

Blocks of conserved synteny have been reported from studies in fungi (e.g. Li *et al*. 2022), plants (e.g. Phan *et al*. 2007) and animals (e.g. Hui *et al*. 2012; Simakov *et al*. 2022). However, this report is the first systematic attempt to document genome-wide synteny in butterflies and moths. It also proposes a simple application of BUSCO analysis as a robust method of defining macrosynteny in any taxa of animals and plants. The conserved synteny blocks in the Lepidoptera, described by the LSU markers, are surprisingly well conserved in the Heteroneura, representing ∼200 (million years) My of conservation. In contrast, macrosynteny decayed rapidly, within ∼100 My, in budding yeast species of the subphylum Saccharomycotina (Li *et al*. 2022) and similarly in nematodes, where Coghlan and Wolfe (2002) detected 2.3–5.4 reciprocal translocations/My in comparisons of the *Caenorhabditis elegans* and *Caenorhabditis briggsae* genomes.

In terms of future applications, LSUs are easily derived from BUSCO analysis of lepidopteran target genomes. They are therefore a simple means to describe the architecture of lepidopteran genomes and to investigate chromosomal evolution. The more complex karyotypes of some lycaenids (blue butterflies) (Lukhtanov 2015) or pierids (white butterflies) (Šíchová *et al*. 2016, Höök *et al*. 2023) are obvious targets for further analysis. Further, BUSCO analysis with LSUs also offers another practical use: to quickly determine places of interest like breakpoints in chromosomal rearrangements for a subsequent detailed analysis of the nature of such breakpoints or fusion points at the nucleotide level. Finally, we predict that this type of homology-based analysis can also be applied to other groups of animals or plants. It will be interesting to determine if the deep conservation of macrosynteny is a specialty of the order Lepidoptera or if other groups of insects show a similar pattern. In this respect, we speculate whether the holokinetic nature of lepidopteran chromosomes compared to the monokinetic nature of chromosomes in most other animal groups is an important factor in their apparent conservation. Understanding and accurately describing macrosynteny is therefore key if we are ever to understand why chromosome numbers vary so considerably in both animals and plants.

## Supplementary Material

jkad134_Supplementary_Data

## Data Availability

All genome data are available in public data banks with acc. nos. given in Supplementary Table 3. Additional details such as Java code and test files for BUSCO output to LSUs are available at Figshare, doi:10.6084/m9.figshare.22672996, for finding and replacement of names (acc. nos.) doi:10.6084/m9.figshare.22673002, and for breakpoint detection doi:10.6084/m9.figshare.22673005. Supplemental material available at G3 online.
