# Supplementary material for: Lepidopteran Synteny Units reveal deep chromosomal conservation in butterflies and moths
Source: G3 (Bethesda). 2023 Jun 13;13(8):jkad134. doi: 10.1093/g3journal/jkad134 (PMC10411566; doi:10.1093/g3journal/jkad134)
Supplement: jkad134_Supplementary_Data [file jkad134_supplementary_data.zip › Supplemental_Material_Legends_G3-2023-404243.docx]

**Supplementary material**

**Table S1** LSU-specific subsets of lepidoptera_odb10 markers.

**Table S2** Genomes used in this study.

Species, family, accession number, chromosome number

**Table S3** Test run.

LSUs represented in *Maniola jurtina*, *Erebia ligea*, and *E. aethiops* chromosomes. The number of markers (Hits) from a specific LSU found on a target chromosome are compared with that on the whole genome (chromosome / genome). Chromosomal relationships found by Pazhenkova and Lukhtanov(2023) and confirmed on an LSU basis are indicated by arrows. Target chromosomes with <4 hits are not listed.

**Table S4** Conservation of LSUs in 13 lepidopteran and one trichopteran species.

The table lists the number of markers from a specific LSU found on a specific chromosome compared with hits on the whole target genome (chromosome / genome). Target chromosomes with <4 hits are not listed.
