# Supplementary material for: Lepidopteran Synteny Units reveal deep chromosomal conservation in butterflies and moths
Source: G3 (Bethesda). 2023 Jun 13;13(8):jkad134. doi: 10.1093/g3journal/jkad134 (PMC10411566; doi:10.1093/g3journal/jkad134)
Supplement: jkad134_Supplementary_Data [file jkad134_supplementary_data.zip › Table_S2_G3-2023-404243.docx]

**Table S2** Genomes used

Species Family accession no. n =

*Biston betularia* Geometridae GCA_905404145.2 31

*Blastobasis adustella* Blastobasidae GCA_907269095.1 30

*Bombyx mori* Bombycidae NC_051358.1 28

- NC_051385.1

*Erebia aethiops* Nymphalidae GCA_923060345.2 20

*Erebia ligea* Nymphalidae GCA_917051295.2 29

*Limnephilus marmoratus* Limnephilidae GCA_917880885.1 30

*Mamestra brassicae* Noctuidae GCA_905163435.1 31

*Maniola jurtina* Nymphalidae GCF_905333055.1 29

*Melitaea cinxia* Nymphalidae GCF_905220565.1 31

*Micropterix aruncella* Micropterigidae GCA_944548615.1 31

*Nematopogon swammerdamellus* Adelidae [OX336333.1](https://www.ncbi.nlm.nih.gov/nuccore/OX336333.1) 31

*--* [OX336363.1](https://www.ncbi.nlm.nih.gov/nuccore/OX336363.1)

*Nymphalis polychloros* Nysmphalidae GCA_905220585.2 31

*Plebejus argus*  Lycaenidae GCA_905404155.2 23

*Plutella xylostella* Plutellidae GCF_932276165.1 31

*Yponomeuta sedellus* Yponomeutidae GCA_934045075.1 31

*Zeuzera pyrina* Cossidae GCA_907165235.1 31

*Zygaena filipendulae* Zygaenidae GCA_907165275.2 30
