# Supplementary material for: Lepidopteran Synteny Units reveal deep chromosomal conservation in butterflies and moths
Source: G3 (Bethesda). 2023 Jun 13;13(8):jkad134. doi: 10.1093/g3journal/jkad134 (PMC10411566; doi:10.1093/g3journal/jkad134)
Supplement: jkad134_Supplementary_Data [file jkad134_supplementary_data.zip › Table_S3_G3-2023-404243.pdf]

LSUs	Maniola jurtina	Hits	Erebia ligea	Hits	Erebia aethiops	Hits
LSU_1	Chr2	293/294	Chr7	270/276	Chr10	295/297
LSU_2	Chr4	292/295	Chr2	296/297	part of Chr5	295/296
LSU_3	Chr1	310/315	Chr1	310/312	Chr11	311/314
LSU_4	Chr6	213/215	Chr5	215/215	Chr13	214/214
LSU_5	Chr3	274/274	Chr4	276/278	Chr12	274/275
LSU_6	Chr7	199/199	Chr6	200/200	part of Chr2	200/204
LSU_7	Chr8	179/186	Chr11	181/182	part of Chr1	180/182
LSU_8	Chr11	223/226	Chr8	223/224	Chr14	223/278
LSU_9	Chr15	224/227	Chr14	224/225	part of Chr4	224/225
LSU_10	Chr14	223/223	Chr10	223/225	part of Chr3	222/275
LSU_11	Chr10	156/156	Chr12	156/158	part of ChrZ	152/159
LSU_12	Chr12	174/175	Chr13	176/176	part of Chr8	175/175
LSU_13	Chr18	136/136	Chr16	136/136	part of Chr3	136/137
LSU_14	Chr9	230/231	Chr15	230/230	Chr16	230/230
LSU_15	Chr17	186/187	Chr19	188/188	Chr17	188/192
LSU_16	Chr16	154/154	Chr18	149/149	part of Chr1	154/155
LSU_17	Chr20	187/189	Chr20	188/191	part of Chr6	187/189
LSU_18	Chr19	184/185	Chr17	184/184	part of Chr7	184/185
LSU_19	Chr21	110/111	Chr22	110/111	part of Chr2	111/112
LSU_20	part of Chr13	162/162	part of Chr9	163/164	part of Chr15	163/164
LSU_21	Chr22	166/167	Chr21	166/166	part of Chr4	168/168
LSU_22	part of Chr5	76/76	Chr3	76/76	part of Chr9	76/76
LSU_23	Chr24	121/121	Chr23	121/121	part of Chr6	121/122
LSU_24	part of Chr5	134/135	Chr3	123/125	part of Chr9	134/135
LSU_25	Chr28	41/41	Chr28	41/42	part of Chr18	42/44
LSU_26	Chr23	53/53	Chr25	53/53	part of Chr7	53/56
LSU_27	Chr26	48/50	Chr24	45/48	part of Chr5	46/49
LSU_28	Chr25	65/65	Chr26	65/65	part of Chr18	65/65
LSU_29	part of Chr27	73/74	Chr27	72/73	part of Chr8	74/76
LSU_30	part of Chr13	46/48	part of Chr9	47/50	part of Chr15	47/51
LSU_31	ChrZ	227/250	ChrZ	227/240	part of ChrZ	228/236
	part of Chr27	4/250				
